# Cesarean Uterine Lacerations and Prematurity in the Following Delivery: A Retrospective Longitudinal Follow-Up Cohort Study

**DOI:** 10.3390/jcm13030749

**Published:** 2024-01-28

**Authors:** Orna Reichman, Ayala Hirsch, Shira Fridman, Sorina Grisaru-Granovsky, Sarit Helman

**Affiliations:** Department of Obstetrics and Gynaecology, Shaare Zedek Medical Centre, Hebrew University, Jerusalem 91120, Israel; ayala46@gmail.com (A.H.); shira.fridman@mail.huji.ac.il (S.F.); sorina@szmc.org.il (S.G.-G.); sarithelman@gmail.com (S.H.)

**Keywords:** second-stage cesarean delivery, lower uterine incision extensions, preterm birth

## Abstract

(1) **Background**: We aimed to investigate whether second-stage cesarean delivery (SSCD) had a higher occurrence of low-segment uterine incision extensions compared with cesarean delivery (CD) at other stages of labor and to study the association of these extensions with preterm birth (PTB). (2) **Methods**: In this retrospective longitudinal follow-up cohort study, spanning from 2006 to 2019, all selected mothers who delivered by CD at first birth (P1) and returned for second birth (P2) were grouped by cesarean stage at P1: planned CD, first-stage CD, or SSCD. Mothers with a PTB at P1, multiple-gestation pregnancies in either P1 or P2 and those with prior abortions were excluded. (3) **Results**: The study included 1574 selected women who underwent a planned CD at P1 (n = 483 (30.7%)), first-stage CD (n = 878 (55.8%), and SSCD (n = 213 (13.5%)). There was a higher occurrence of low-segment uterine incision extensions among SSCD patients compared to first-stage CDs and planned CDs: 50/213 (23%), 56/878 (6.4%), and 5/483 (1%), respectively (*p* < 0.001). A multivariate logistic regression showed that women undergoing an SSCD are at risk for low-segment uterine incision extensions compared with women undergoing a planned CD, OR 28.8 (CI 11.2; 74.4). We observed no association between the occurrence of a low-segment uterine incisional extension at P1 and PTB ≤ 37 gestational weeks in the subsequent delivery, with rates of 6.3% (7/111) for those with an extension compared to 4.5% (67/1463) for those without an extension (*p* = 0.41). Notably, parturients experiencing a low-segment uterine incisional extension during their first childbirth were six times more likely to have a preterm delivery before 32 weeks of gestation compared to those without extensions, with two cases (1.8%) compared to four cases (0.3%), respectively. A similar trend was observed for preterm deliveries between 32 and 34 weeks of gestation, with those having extensions showing twice the prevalence of prematurity compared to those without, with a *p*-value of 0.047. (4) **Conclusions**: This study highlights that mothers undergoing SSCD experience higher prevalence of low uterine incision extensions compared to other CDs. To further ascertain whether the presence of these extensions is associated with preterm birth (PTB) in subsequent births, particularly early PTB before 34 weeks of gestation, larger-scale future studies are warranted.

## 1. Introduction

Preterm birth (PTB), defined as delivery before the completion of 37 weeks of gestation, complicates 12–13% of deliveries in the United States and 5–9% of deliveries in Europe. It is the primary cause of long-term morbidity during the perinatal period, contributing to 75% of perinatal deaths, and it is dependent on various factors, such as the level of prematurity, timing of birth (early or late preterm), and underlying pathologies [1]. As a multifactorial phenomenon, PTB is associated with various factors, including maternal infection, inflammation, uteroplacental disorders, uterine overdistension, immunologically mediated processes, and a history of a previous PTB [2].

Few studies have demonstrated the association between a second-stage cesarean delivery (SSCD) and PTB in the following delivery [3,4,5,6,7]. The hypotheses proposed for this association include structural cervical trauma by various mechanisms: (1) an unintended incision of the cervix or internal os at the time of full dilatation—misidentifying the lower uterine segment, (2) an unintended inclusion of cervical structures in the closure of a uterine incision, and (3) inadvertent extensions of a lower uterine incision, presumed to occur more often among SSCDs compared with planned or first-stage cesarean deliveries (CDs) [8,9]. This structural cervical trauma can potentially damage the muscle body of the internal os and cause cervical dysfunction in future pregnancies, resulting in cervical incompetence and PTB [8,9,10].

We aimed to study the association between lower uterine incision extensions during SSCD compared with CD at other stages of labor and their association with preterm birth in the following delivery.

## 2. Materials and Methods

A retrospective longitudinal follow-up cohort study was conducted in a single large tertiary university hospital, Shaare Zedek Medical Center (SZMC), Jerusalem, Israel between January 2006 and December 2019.

Study population: The obstetric setting of the study population was described in detail [11]. Briefly, approximately 14,500 deliveries occur annually at the study site, of which approximately 25% are nulliparous, 18% are grand multiparous (parity ≥ 6), 12% are cesarean deliveries, and 5% are vacuum deliveries. Delivery records of women who delivered both first (P1) and second (P2) children at SZMC were screened. All mothers with a CD at first delivery were included, excluding those with preterm delivery at P1, parturients with multiple gestation at either delivery, and those with previous abortions. We chose not to exclude cases involving a malformed uterus, despite their association with PTB, as there is a potential association to low-segment uterine incisional extensions. Selected mothers were grouped according to the stage of CD at P1: (1) planned CD, (2) first-stage CD, and (3) SSCD.

Data collection: Data were extracted from a computerized database that is continually updated by the staff attending the labor and delivery. Most of the variables in the medical record were fixed, and mandatory fields were required to be completed before transfer from the delivery ward. Data retrieved for both P1 and P2 included baseline obstetric characteristics: maternal age, gestational age at delivery, onset of labor (spontaneous, induction, or planned CD), stage of labor of the CD at P1 (planned, first stage, or SSCD), low-segment uterine incisional extensions at P1, interpregnancy interval, birthweight of newborn, Apgar score at 1 and 5 min, and admission ward of the newborn. A low-segment uterine incisional extension was defined as an additional tear in the low-segment tissue after the initial uterine excision. However, excisions involving the body of the uterus, which may prevent a trial of vaginal birth following a CD, were excluded from the analysis. The presence of a low-segment uterine incisional extension is a non-mandatory field in the electronic medical record; therefore, all surgical notes of P1 were screened for the word “extension” and read thoroughly to verify if a low-segment uterine incisional extension occurred during the CD.

Sample Size: Annually, there were approximately 14,500 births at SZMC; thus, over the 14-year study period, there would be 200,000 births, of which 50,000 (~25%) were primiparas. Based on prior data, a third of primiparas do not return to SZMC for the second delivery [12]. Thus, only 33,000 (66% of 50,000) were expected to return for the second birth. Therefore, it was estimated that 3960 mothers delivered both first (P1) and second (P2) deliveries at SZMC, with the first delivery culminating in a CD (33,000 × 0.12 {CD rate}). After the exclusion of multiple gestations or those with prior miscarriages and medical records with missing data, it was estimated that 2000 mothers would be included in the current study.

Statistics: Data were validated by defining distributions and quantifying missing values. Obstetric characteristics comparing the three CDs groups were presented as proportion or mean with standard deviation, depending on the variable characteristics: categorical or continuous, respectively. Statistical significance was defined by a two-sided *p*-value ≤ 0.05 using the chi-square test or Fisher exact test for categorical variables. Analyses were performed using IBM SPSS ^®^ Statistics (version 29).

The study was approved by the Institutional Review Board of the Shaare Zedek Medical Center (SZMC IRB 0100-19). As this was a retrospective study, a waiver of informed consent was obtained. The manuscript is presented according to the STROBE guidelines [13].

## 3. Results

**Figure 1:**
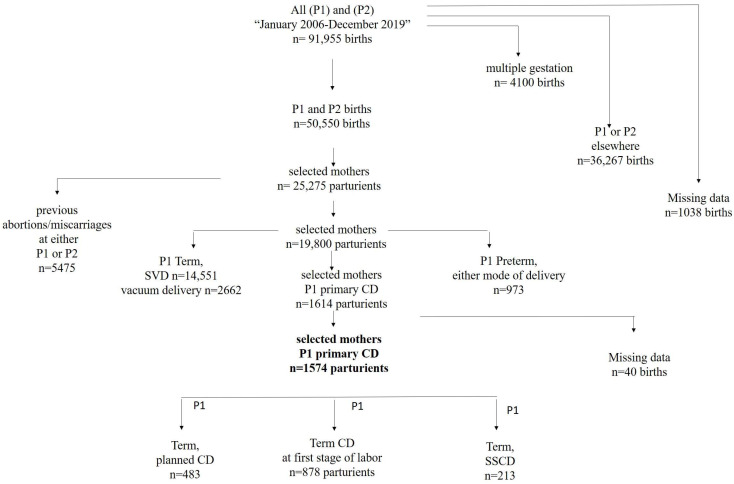
Flowchart of study population.

The initial search identified 91,955 women who delivered the first and/or second delivery at SZMC. After excluding mothers who delivered elsewhere at either first (P1) or second (P2) delivery, those with previous abortions/miscarriages, those with a history of multiple pregnancies, and those with missing data, a total of 19,800 mothers were identified. Of these, excluding women with a vaginal delivery and/or preterm birth at P1 left a sample of 1574 women that underwent a CD at P1: 483 (30.7%) had a planned CD, 878 (55.8%) had a first-stage CD, and 213 (13.5%) had an SSCD, as shown in Figure 1.

Low-segment uterine incision extensions occurred more often in SSCDs compared to first-stage CDs and planned CDs, 50/213 (23%), 56/878 (6.4%), 5/483 (1%), *p* < 0.001, respectively. Prematurity in the following delivery did not differ significantly between the groups and ranged from 4.1 to 5.7%, *p* = 0.367. The gender of newborns, macrosomia (>4000 g), and neonatal intensive care unit (NICU) admissions differed significantly between the groups. The data are presented in Table 1.

A multivariate logistic regression analysis including maternal age at P1, birthweight of newborn at P1, and stage of CD at P1 (planned, first stage, or second stage of labor) found that women undergoing an SSCD are at increased risk for lower uterine incision extensions, with an OR of 28.8 (CI 11.2; 74.4), compared with women undergoing a planned CD and with women undergoing a CD at the first stage of labor, OR 4.3 (CI 2.8; 6.6).

A binary logistic regression model searching for factors associated with PTB was conducted. Of the factors studied at first birth, including SSCD, low-segment uterine incisional extension, duration of the second stage, duration of cesarean operation, breech presentation, blood transfusion, and birthweight of the newborn, none were found to be associated with PTB, as shown in Table 2. Maternal age at second birth was found to be associated with PTB, 1.066 (95% CI 1.023; 1.110, *p* = 0.002). Interpregnancy interval was not found to be associated with PTB 0.846 (95% CI 0.492; 1.456, *p* = 0.546). The data are presented in Table 2.

We observed no association between the occurrence of a low-segment uterine incisional extension at P1 and PTB ≤ 37 gestational weeks in the subsequent delivery, with rates of 6.3% (7/111) for those with an extension compared to 4.5% (67/1463) for those without an extension (*p* = 0.41). Stratifying the groups by the occurrence of a low-segment uterine incision extension revealed that secundiparas with a history of SSCD with a low-segment uterine incision extension were at an increased risk for PTB, although statistically non-significant, compared to secundiparas without a lower uterine incision extension: 4/50 (8%), 6/163 (3.7%), *p* = 0.185, respectively. The data are presented in Table 3.

Additionally, we conducted further analysis to explore the association between the occurrence of a low-segment uterine incisional extension at P1 and prematurity, categorized by gestational weeks: ≤31.6 weeks, 32.0–34.0 weeks, 35.0–36.0 weeks, and ≥37.0 weeks. Our findings indicate that parturients experiencing a low-segment uterine incisional extension during their first childbirth were six times more likely to have a PTB before 32 weeks of gestation compared to those without extensions. Specifically, the incidence was two cases (1.8%) among parturients with extensions compared to four cases (0.3%) among those without. A similar trend was observed for PTB between 32 and 34 weeks of gestation, with those having extensions showing twice the prevalence of prematurity compared to those without, *p* = 0.047. Detailed data are provided in Table 4.

Initially, our objective was to record the diverse indications for CD and examine whether there was an association between specific indications and low-segment uterine incisional extensions. Consequently, we scrutinized all the diagnoses documented in the summary notes of the CDs. Unfortunately, not all of the listed diagnoses were genuine indications for CD, such as chorioamnionitis and premature rupture of the membrane. Consequently, we explored the potential associations between various pathologies and the occurrence of a low-segment uterine incisional extension. Initially, we performed a chi-square analysis to explore the associations between the various pathologies and the stage of cesarean—planned, first, or SSCD. The data are presented in Table 5.

All eight defined pathologies were clinically and statistically significantly associated with the timing of the CD. The diagnosis of ‘fetal distress’ was much more common for CDs at the first stage of labor (n = 385, 43.8%) compared with planned CDs (n = 40, 8.3%) and SSCDs (n = 54, 25.4%), *p* < 0.001. The diagnoses of ‘Intrapartum fever/chorioamnionitis’ were comparable in the first and second stage of labor, with 63 (7.2%) vs. 16 (7.5%), respectively, but were very rare in planned CDs, with 2 (0.4%), *p* < 0.001. ‘Failed vacuum extraction’, as expected, occurred only with SSCDs, with 58 (27.2%), *p* < 0.001. On the other hand, ‘breech presentation’ was much more common during planned CDs compared with the first and the second stage of labor, with 198 (41.8%), 51 (5.8%), and 2 (1%), respectively, *p* < 0.001).

The associations between the pathologies studied and the occurrence of a low-segment uterine incisional extension are depicted in Table 6. Fetal distress, intrapartum fever/chorioamnionitis, failed vacuum delivery, and breech presentation were significantly associated with a low-segment uterine incisional extension. In contrast, arrest of dilatation, malformed uterus, pregnancy-induced hypertension, and premature rupture of membranes were not associated with low-segment uterine incisional extension, as shown in Table 6.

Regrettably, our dataset lacks information regarding the time elapsed from the decision to proceed with a cesarean delivery (CD) to the actual delivery. Consequently, we could not explore the relationship between the time from decision-making to uterine incision and the presence of low-segment uterine incisional extensions. However, we did have access to data regarding the CD duration. Upon comparing the mean CD duration between cases with low-segment uterine incisional extension and those without, we discovered a clinically significant positive association. Cases with extensions had a mean CD duration of 42.95 min, while those without extensions had a mean duration of 29.50 min, indicating a considerable mean difference of 13.45 min (*p* < 0.001). This finding aligns with the expected scenario, as surgeries involving incisional extensions often demand extra time for suturing and repair.

## 4. Discussion

The current study highlights that parturients undergoing an SSCD are at increased risk for low-segment uterine incisional extensions compared with planned CDs, OR 28.8 (CI 11.2; 74.4), and with first-stage CDs, OR 4.3 (CI 2.8; 6.6). This finding is consistent with a cohort study published two decades ago in Bristol, UK, of 209 parturients undergoing SSCDs that reported low-segment uterine incisional extensions in 24% (50/209) [14], similarly to the percentage reported in the current study: 50/213 (23.5%). This was also reported in a study from Israel, published a decade ago, which compared the prevalence of low-segment uterine incisional extension between the first (n = 306) and second (n = 76) stages of CD, with 4.6% versus 17.1%, respectively [15], which is comparable to the OR of 4.5 that we reported in the current study. These reports are consistent with the findings of a recent study comparing uterine extension occurring in first-stage CDs, 42/707 (6.2%), with SSCDs, 52/329 (16.8%), in Warwickshire, UK [16]. Thus, together with the current study, it is well established that women undergoing an SSCD are at risk for low-segment uterine incisional extension.

It is proposed that low-segment uterine incisional extension can potentially cause mechanical injury to the internal os [8,9,10]. A new paradigm suggests that the smooth muscle cells in the internal os of the human cervix function as a sphincter. This assumption is based on analyzing 41 cervical tissues of non-pregnant women undergoing hysterectomies for benign indications and describing the presence of smooth muscles and connective tissue. The study revealed that the internal os contains approximately 50–60% smooth muscle tissue that is organized circumferentially around the endocervical canal, compared with the external os, which is built mostly by collagen tissue, with only 10% smooth muscle cells [17]. Mechanical injury to the internal os, which functions similarly to a sphincter, could potentially lead to impairment of cervical function in future pregnancies, resulting in cervical incompetence and PTB [8,9,10].

Previous studies searched for an association between SSCD and PTB based on the understanding that SSCD is associated with low-segment uterine incisional extension and that uterine extensions can cause mechanical injury to the cervix, possibly leading to cervical incompetence and PTB [3,4,5,6,7,11,16,18,19,20]. Some found a positive association [4,5,18,19,21] and others did not find an association [7,16,20,22]. Most of these reports studied the association between SSCD and PTB [3,4,5,7,11,18,19] or SSCD and low-segment uterine incision extension [4,20] with only one of these studies combined all three [16]. Data are presented in Table 7.

To our knowledge, the current study and the Ewington et al. historical cohort study from UK [16] are the only two studies that have documented low-segment uterine incisional extensions from the surgical notes of the SSCDs that directly preceded the PTBs.

No association was found in the current study between the timing of CD—planned, first-stage, or SSCD—and PTB in the following delivery, with 28/483 (5.7%), 36/878 (4.1%), and 10/213 (4.7%), respectively, *p* = 0.367. These findings are similar to the results of Ewington et al., who compared CDs at three stages of cervical dilatation: 0–5 cm (n = 707), 6–9 cm (n = 423), and SSCD (n = 329) with PTB in the subsequent delivery. They reported PTBs among 25/707 (3.5%), 17/423 (4.0%), and 17/329 (5.2%), *p* = (0.22–0.68), deliveries, which is comparable with the findings of the current study [16].

The current study did not find an association between factors from first birth and PTB in the following delivery, including SSCD versus planned CD, low-segment uterine incisional extensions, duration of the second stage, breech presentation, duration of cesarean operation, birthweight of newborn, or need for maternal blood transfusion. A study by Ewington et al. also failed to show an association between SSCD, newborn birthweight, and low-segment uterine incisional extensions at first birth with subsequent PTB. Of note, the study by Ewington et al. found a positive association between interpregnancy interval (categorical variable, less than one year) and PTB, aOR of 3.10 (1.71–5.61), *p* < 0.001, in contrast to the current study that failed to find such an association, OR 0.846 (0.492; 1.456), *p* = 0.546, possibly as a result of the unselected inclusion criteria of the study population [16]. The current study included only mothers with first and second birth, excluding previous abortions/miscarriages, as opposed to the study by Ewington et al., which included mothers with parity ≥2 and a history of late miscarriages and previous CD prior to the indexed CD. It is important to highlight that the present study has identified a positive association between an SSCD following a failed vacuum to a low-segment uterine segment extension. This observation aligns with a previous study conducted by our group, which reported a positive association between an SSCD following a failed vacuum and PTB [11]. Stratifying the groups of CDs by the occurrence of a low-segment uterine incisional extension did not show an association between secundiparas with a history of SSCD with extension compared with secundiparas without uterine extensions, RR 2.3 (CI 0.6; 8.4, *p* = 0.218). It is important to note that the small sample size in both groups, SSCD with/without extension (n = 50, n = 167, respectively), renders the analysis underpowered. The calculated statistical power to achieve significance indicates that 467 participants are needed in each group.

A noteworthy discovery is the observed association between low-segment uterine incisional extensions and prematurity before 34 weeks of gestation in subsequent deliveries. Despite the relatively modest sample size, the clinical significance stands out, revealing a sixfold higher prevalence of prematurity prior to 32 weeks of gestation in the subsequent delivery among parturients with extensions compared to those without. Importantly, it is observed that this association does not extend to late preterm birth (35–36 weeks of gestation). A plausible explanation for this observation could be that the pathologies contributing to prematurity are associated with the timing of prematurity, distinguishing between early and late occurrences.

Strengths of the study include the following: (1) The current study design excluded known factors with a strong association with PTB, such as previous PTB, multiple pregnancy, and previous miscarriage. (2) By including only selected primiparas and secundiparas, the effect of parity was neutralized. (3) Many of the confounding factors associated with PTB, such as socioeconomic status, BMI, genetic factors, chronic illness, smoking, and alcohol habits, that were not available or missing from the dataset were neutralized, as each woman served as her own control. (4) The majority of variables analyzed are mandatory fields in the electronic medical record and were, therefore, accessible. (5) Comparable findings to other studies strengthen the study’s external validity. (6) Although the identification of low-segment uterine incisional extensions relied on non-mandatory fields extracted from computerized searches of free text in surgical notes in the electronic medical records, the anticipated clinical outcome of prolonged CD duration in cases with low-segment uterine incisional extensions implies the absence of recall bias or missing data, thereby strengthening the study’s internal validity.

The current study has several limitations: (1) Known factors that are associated with PTB, including urinary tract infection, placental abruption or circumvallate placenta, previous cervical conization or LEEP excision, and systemic infections, were missing and, therefore, not able to be adjusted for in the regression analysis. (2) Single-center studies are homogenous by nature, unlike multicenter studies with potential differences in obstetric management and treatment protocols, which could affect the study’s external validity. However, strict inclusion criteria of a narrowly defined group of parturients with similar characteristics described by others minimize the significance of this limitation. (3) The disadvantage of a small sample size leads to the possibility of the study being underpowered to show a significant association, as we discussed regarding the finding of secundiparas with a history of SSCD with extension having increased risk for PTB compared with secundiparas without such a complication, RR 2.3 (CI 0.6; 8.4, *p* = 0.218). Future studies are needed to verify if our assumption concerning the lack of significance due to the limited sample size is correct. (4) An impacted fetal head is estimated to complicate one in ten unplanned CDs and is associated with low-segment uterine incision extensions. There are several techniques to assist the delivery of a baby with an impacted head, and these include an assistant applying pressure to reposition the baby’s head from the vaginal canal, delivering the baby in a feet-first position, employing a specifically designed inflatable balloon device to raise the baby’s head, and/or administering medication to relax the womb [23]. There is no consensus on which maneuver is best for reducing complications, yet documenting the technique used could have contributed to our knowledge regarding the association between the maneuver used and unintended low-segment uterine extensions. (5) Subsequently, it is essential to acknowledge that we lacked data regarding the level of the head prior to delivery, which is a potential factor associated with low-segment uterine incision extensions. (6) Despite a thorough examination of all surgical notes related to P1 and excluding those involving the body of the uterus from the analysis, the data lack a comprehensive description of the size and length of the extension, which theoretically could be associated with the studied outcome of PTB.

## 5. Conclusions

Women undergoing an SSCD are at increased risk for low-segment uterine incisional extensions. The association between SSCD and PTB is unclear, given the inconsistent findings of studies. However, our study demonstrated an association with early PTB before 34 weeks of gestation, implying that the pathologies contributing to prematurity are associated with the timing of prematurity, distinguishing between early and late occurrences. To further ascertain whether the presence of these extensions is associated with preterm birth (PTB) in subsequent births, particularly early PTB before 34 weeks of gestation, larger-scale future studies are warranted.

## Figures and Tables

**Table 1:** Obstetric characteristics of 1574 parturients grouped by timing of CD at first delivery ^1^.

		P1 Term Planned CD N = 483	P1 Term First-Stage CD N = 878	P1 Term SSCD N = 213	*p*-Value
P1 first delivery					
P1 maternal age, years, mean ± std		26.4 ± 5.4	25.8 ± 4.6	25.8 ± 4.6	0.002
P1 macrosomia (>4000 g), N		46 (9.5%)	47 (5.4%)	23 (10.9%)	0.002
P1 female gender		249 (51.6%)	372 (42.2%)	79 (37.1%)	<0.001
P1 Apgar at 5 min ≤ 7		6 (1.2%)	27 (3.1%)	5 (2.3%)	0.108
P1 NICU		13 (2.7%)	7 (4.4%)	18 (8.5%)	0.004
P1 unintended uterine extension		5 (1.0%)	56 (6.4%)	50 (23.5%)	<0.001
P2 second delivery					
Onset of labor	Spontaneous	265 (54.9%)	521 (59.3%)	156 (73.2%)	
	Induction	30 (6.2%)	76 (8.7%)	9 (4.2%)	<0.001
	CD—no trial of labor	188 (38.9%)	281(32.0%)	48 (22.5%)	
Mode of delivery	Spontaneous	189 (39.1%)	319 (36.3%)	84 (39.4%)	
	Vacuum extraction	39 (8.1%)	99 (11.3%)	37 (17.4%)	0.009
	CD	255 (52.8%)	458 (52.2%)	92 (43.2%)	
Preterm birth prior to 37 weeks of gestation		28 (5.7%)	36 (4.1%)	10 (4.7%)	0.367

^1^ Excluding parturients with a history of abortions and multiple gestations.

**Table 2:** Parameters explored by univariate regression analysis for an association with preterm birth in the second pregnancy.

	OR (95% CI)	*p*
**Factors at P1**		
SSCD versus planned CD	0.800 (0.383; 1.679)	0.556
Low-segment uterine incisional extension	1.402 (0.628; 3.132)	0.409
Duration of the second stage	1.000 (0.997; 1.004)	0.815
Breech presentation	1.048 (0.608; 1.807)	0.865
Duration of cesarean operation	1.000 (0.996; 1.004)	0.862
Birthweight of newborn	1.000 (0.999; 1.000)	0.061
Blood transfusion	2.493 (0.735; 8.453)	0.143
**Factors at P2**		
Maternal age	1.066 (1.023; 1.110)	0.002
Interpregnancy interval of less than one year	0.846 (0.492; 1.456)	0.546

Second-stage cesarean delivery (SSCD), cesarean delivery (CD), odds ratio (OR).

**Table 3:** The associations between the timing of CD at first delivery and PTB at the following delivery.

Timing of CD at First Delivery (P1)	Status of Extensions in CD at P1	Preterm Delivery P2	*p*-Value
Planned CD	No extension (n = 478)	28 (5.9%)	0.741
	With extension (n = 5)	0	
First-stage CD	No extension (n = 822)	33 (4%)	0.407
	With extension (n = 56)	3 (5.4%)	
SSCD	No extension (n = 163)	6 (3.7%)	0.185
	With extension (n = 50)	4 (8%)	

Second-stage cesarean delivery (SSCD), cesarean delivery (CD), preterm birth (PTB).

**Table 4:** The associations between low-segment uterine incisional extension at first birth (P1) to prematurity at the following delivery (P2), grouped by gestational week among 1574 parturients undergoing a cesarean delivery at first birth ^1^.

		P2 Preterm Birth				*p*-Value
		≤31.6 Weeks n = 6	32–34 Weeks n = 13	35–36 Weeks n = 55	≥37.0 Weeks n = 1500	
P1 cesarean delivery n (%)	Incisional extensions 111 (7%)	2 (1.8%)	2 (1.8%)	3 (2.7%)	104 (93.7%)	0.047
	No extensions 1463 (93%)	4 (0.3%)	11 (0.8%)	52 (3.6%)	1396 (95.4%)	

^1^ Excluding parturients with a history of abortions and multiple gestations.

**Table 5:** Various pathologies of 1574 parturients grouped by timing of first cesarean delivery ^1^.

	P1 Term Planned CD N = 483	P1 Term First-Stage CD N= 878	P1 Term SSCD N = 213	P1 Term Total N = 1574	*p*-Value
Fetal distress *	40 (8.3%)	385 (43.8%)	54 (25.4%)	479 (30.4%)	<0.001
Intrapartum fever/chorioamnionitis	2 (0.4%)	63 (7.2%)	16 (7.5%)	81 (5.1%)	<0.001
Malformed uterus	20 (4.1%)	23 (2.6%)	0	43 (2.7%)	0.008
Gestational hypertension **	4 (0.8%)	43 (4.9%)	7 (3.3%)	54 (3.4%)	<0.001
Failed vacuum	0	0	58 (27.2%)	58 (3.7%)	<0.001
Arrest of dilatation	1 (0.2%)	85 (9.7%)	8 (3.8%)	94 (6%)	<0.001
Premature rupture of membranes	45 (9.5%)	154 (17.6%)	28 (13.3%)	227 (14.6%)	<0.001
Breech presentation	198 (41.8%)	51 (5.8%)	2 (1%)	251 (16.1%)	<0.001

^1^ Parturients could have more than one diagnosis. * Fetal distress was defined as one of the following diagnoses: fetal distress, variable deceleration, abruption, cord prolapse, occult cord, or meconium. ** Preeclampsia and gestational hypertension.

**Table 6:** The association between various pathologies and the occurrence of low-segment uterine incision extensions among 1574 parturients undergoing a cesarean delivery at first birth ^1^.

	No Extension n = 1463	Low-Segment Uterine Incision Extensions n = 111	*p*-Value
Fetal distress *	435 (29.7%)	44 (39.6%)	0.020
Intrapartum fever/chorioamnionitis	65 (4.4%)	16 (14.4%)	<0.001
Malformed uterus	40 (2.7%)	3 (2.7%)	0.640
Pregnancy-induced hypertension **	50 (3.4%)	4 (3.6%)	0.537
Failed vacuum	45 (3.1%)	13 (11.7%)	<0.001
Arrest of dilatation	88 (6.1%)	6 (5.5%)	0.498
Premature rupture of membranes	211 (14.6%)	16 (4.5%)	0.563
Breech presentation	246 (17.0%)	5 (4.5%)	<0.001

^1^ Excluding parturients with a history of abortions and multiple gestations. * Including cases with meconium, abruption, cord prolapse, non-reassuring fetal monitor; ** Including cases with either preeclampsia or hypertension

**Table 7:** Previous studies that focused on the association of SSCD and low-segment uterine incisional extensions and their associations with preterm birth.

Reference First Author [# Ref]	SSCD Number	SSCD Low-Segment Uterine Incisional Extensions n (%)	Association between SSCD and PTB (OR)	PTB Subsequent to Uterine Incisional Extensions (OR/Prevalence)
Wood, S.L., et al. [3]	8607	-	1.57 (1.43; 1.73) *	-
Levine, L.D., et al. [4]	37	-	2.40 (0.77–7.43) *	13.5% #
			5.80 (1.08–30.80) **	
Watson, H.A., et al. [5]	29	-	3.06 (1.22–7.71) *	-
Cong, A., et al. [7]	533	-	1.50 (0.97–2.20) **	-
Helman, S., et al. [11]	221	-	3.8% vs. 3.4% *	-
Murphy, D.J., et al. [14]	209	50 (24%)		
Lurie, S., et al. [15]	76	13 (17.1%)		-
Ewington, L.J., et al. [16]	329	55 (16.8%) ^^	1.86 (0.91–3.83) **	1.34 (0.54–3.29)
Williams, C., et al. [18]	483	-	3.29 (2.02–5.13) *	-
Offringa, Y., et al. [19]	143	-	2.50 (1.30–4.90) *	-
Levine, L.D., et al. [20]	37	not documented	2.08 (0.32–13.78) *	20.0% ^
Reichman, O., et al. [Current study]	213	50 (23.5%)	0.800 (0.38; 1.68) ***	1.402 (0.628; 3.132)

Second-stage cesarean delivery (SSCD), preterm birth (PTB), odds ratio (OR); * reference vaginal delivery/** reference first-stage CD/*** reference planned CD; # women with an extension at the time of cesarean (n = 38), regardless of stage of labor at which the cesarean was performed, were more likely to have a spontaneous PTB compared with women without an extension (n = 128); ^ underpowered, with only 37 women with SSCDs; ^^ low-segment uterine incision extensions in SSCD compared to CD at 0–5 cm.

## Data Availability

Data are available upon reasonable request from the corresponding author.

## Abbreviations

Cesarean delivery (CD), second-stage cesarean delivery (SSCD), preterm birth (PTB), first birth (P1), second birth (P2), odds ratio (OR), adjusted odds ratio (aOR), Shaare Zedek Medical Center (SZMC), neonatal intensive care unit (NICU), relative risk (RR), confidence interval (CI), body mass index (BMI), loop electrosurgical excision procedure (LEEP)
